# Uncertainties and uncertain risks of emerging biotechnology applications: A social learning workshop for stakeholder communication

**DOI:** 10.3389/fbioe.2022.946526

**Published:** 2022-09-27

**Authors:** Britte Bouchaut, Huib de Vriend, Lotte Asveld

**Affiliations:** ^1^ Delft University of Technology, Delft, Netherlands; ^2^ LIS Consult, Driebergen, Netherlands

**Keywords:** uncertain risks, safety, mutual learning, safe-by-design, responsibility, plant engineering

## Abstract

Emerging applications of biotechnology such as new genomic techniques may give rise to new uncertainties and uncertain risks. Particularly the increased complexity and limited knowledge of possible risks associated with these new techniques, make it currently impossible to perform an adequate environmental risk assessment. As a result, there is a risk that such techniques don’t get beyond experiments demonstrating the proof of principle, stifling their further development and implementation. To break free from this deadlock, we must be able to learn what such uncertainties and uncertain risks entail, and how they should be assessed to ensure safe further development. To shape a responsible learning environment to explore uncertainties and uncertain risks, we have organized five stakeholder workshops. By means of a case about the genetic engineering of plants’ rhizosphere–an application abundant with uncertain risks–we identified tensions between different stakeholder groups and their different estimates of uncertainties and uncertain risks. Based upon derived insights, we developed a tool–a script for researchers to organize a stakeholder workshop–that enables a constructive discussion about emerging risks with a broad range of stakeholders. Thereby, the script provides a step-by-step approach to identify uncertainties, develop anticipatory strategies and adaptations in (experimental) research designs to lower or mitigate the earlier identified uncertainties, and helps to identify knowledge gaps for which (additional) risk research should be set up.

## 1 Introduction

Already in 2017, Hogervorst and colleagues pointed out various developments in biotechnology for which no adequate environmental risk assessment (ERA) could be performed at that moment (Hogervorst et al., 2017). In particular, the increasing complexity associated with new genomic techniques, and lack of knowledge thereof, gives rise to debate on how to execute an ERA in such cases (Parisi and Rodriguez Cerezo, 2021). Currently, Europe’s risk management regime regarding biotechnology seems to be one of compliance (Bouchaut and Asveld, 2021), which provides little room to learn what uncertainties and uncertain risks entail. With these types of uncertainty, we refer to the so-called “known unknowns”—instances of which we know we are missing information about the probability or severity of a harmful effect, or of which we do not know if there are any possible harmful effects to begin with (Aven and Renn, 2009). Due to the strong embeddedness and operationalization of the precautionary principle (PP), potentially having a risk involved is sufficient to take cost-effective measures to prevent environmental degradation. In other words; uncertainty does not justify inaction, or ultimately limits research (Brisman, 2011). However, these measures should be based on an examination of the potential benefits and costs, or lack of action, and be subject to review in the light of new scientific data (Commission of the European Communities, 2000).

The way the PP has been operationalized in Europe has resulted in a normative framework in which the biological safety protocol is currently subjected to a dilemma between safety and innovation. While it ensures safety on known and acceptable risks, it also hinders innovation as it stifles research with uncertainties involved. Indeed, present regulation based on the PP only allows very little room for learning about uncertainties and how to mitigate uncertain risks, and thus also whether uncertainties should be regarded as uncertain risks, and uncertain risks as (unacceptable) risks (van Asselt and Vos, 2006, van Asselt and Vos, 2008; Flage and Aven, 2015). In addition, learning being limited also results in maintaining a lack of knowledge regarding the potential benefits, which also creates a deadlock for reviewing earlier taken precautionary measures in the light of new knowledge.

To break free from this impasse, the process of ERA must create more room to learn what uncertainties and uncertain risks entail, and based on this information, define how to assess and regard these. But this learning may be complicated by differing perspectives from stakeholders on uncertainties and uncertain risks (Bouchaut and Asveld, 2020). So foremost, we need to increase mutual understanding of differing perspectives. A new approach to facilitating learning about uncertainties (both potential risks and benefits) would require extensive communication and mutual learning between various stakeholders. Although dependent on the partaking stakeholders’ fields of expertise (e.g., technical, regulatory or societal domain), we must ensure that this learning is conducted in line with possible (societal) concerns and that any results are taken up swiftly by relevant stakeholders to allow for some form of adaptive risk management. The question that emerges from this is how to organize such a learning process with a variety of stakeholders. This paper’s aim is therefore twofold: develop a tool that enables a learning process regarding emerging uncertain risks and uncertainties, and evaluate whether learning has occurred. To do so, we organized five stakeholder workshops with participants from a range of expertise (e.g., technical researchers, social scientists, risk assessors, policymakers), building upon the International Risk Governance Council (IRGC) framework and the notion of “social learning” by van de Poel (2017).

The importance of learning processes is acknowledged by the IRGC framework that provides guidelines for dealing with situations characterized by a mix of complexity, uncertainty and/or normative ambiguity (Renn and Walker, 2008; IRGC, 2017). Particularly the framework’s first step, the pre-assessment, involves relevant stakeholder groups to capture differing perspectives on potential risks, their associated opportunities and potential strategies to address these (IRGC, 2017). For our workshops, we complemented the IRGC’s pre-assessment with three levels of “social learning” about uncertainties and relevant technical, governmental and societal aspects (van de Poel, 2017). These levels are 1) *impact learning*, which addresses uncertainties associated with the social impacts of a new technology, which can be both positive and negative; 2) *normative learning*, referring to what “we” think would be desirable or not and calls f or a balance between ensuring safety and being able to take some risk to gain knowledge of uncertainties; and 3) *institutional learning* addressing responsibility allocation, e.g., who decides what risk would be acceptable? And who establishes norms?

During the workshops, we made use of a case study that focuses on an emerging biotechnology application with several associated uncertainties and uncertain risks. Through this case and implementing the three levels of social learning, the discussions conducted in the workshops provided insights into tensions between the partaking stakeholder groups in terms of how to manage uncertainties and uncertain risks, what would be needed to overcome these tensions, and what would be needed to organize a learning process about these potential risks from emerging biotechnology applications? Based on these insights, we developed a tool–a script and guidelines–for researchers to organize a stakeholder workshop that enables a suitable environment in which learning processes can take place. Via this learning process, a range of partaking stakeholders can collectively identify different estimates of emerging risks and develop anticipatory strategies to lower or mitigate these. As a result, adaptations in (experimental) research designs can be defined to ensure safety, and knowledge gaps are identified for which complementary risk research should be set up.

## 2 Materials and methods

A total of five workshops were conducted; one in March 2021, two in June 2021, one in January 2022 and one in February 2022. Due to COVID-19, all workshops were conducted in an online environment with a maximum duration of 2.5 h. From all workshops, an anonymized transcript was made which was coded and analyzed accordingly. Prior to all workshops, participants signed a form giving consent to record the workshop (audio and video). Furthermore, of the five workshops, two (March and June 2021) were held in English, and three (June 2021, January 2022 and February 2022) were conducted in Dutch as all participants in these workshops were native Dutch-speaking. Therefore, quotes from these latter three workshops have been translated into English. All transcripts and original quotations are available upon request from the corresponding author^1^.

### 2.1 Research design

There is a need for a constructive discussion about emerging risks and how to assess them/ learn about them responsibly. Using a case study, which is elaborated on in the next section, we first wanted to identify tensions between stakeholder groups that might complicate further communication and knowledge exchange between these groups. This mostly pertained to differing perspectives on emerging uncertainties and differences in the acceptability of these, possibly causing difficulty in progressing with experimental research safely and responsibly. All workshops were dedicated to gaining such insights.

As already mentioned, the workshops were built upon the pre-assessment step within the IRGC framework. But, to make this step more concrete for our workshop and to gain a more holistic approach, we have implemented the notion of social learning. Particularly its three levels of learning about uncertainties, namely: normative, impact and institutional (van de Poel, 2017). In the two workshops conducted in March and June 2021, a (plenary) discussion was devoted to each level of learning. Table 1 provides an overview of the organization of these workshops in the form of a short script. For the next “normative learning” step, we made use of an online discussion platform (i.e., ConceptBoard) that would make this step more interactive. Within the workshop, participants were divided into two “break-out” sessions and each was moderated either by MOD or by one of the present observers (OBS).

**TABLE 1:** Script for Workshops conducted in March and June 2021. MOD, Moderator of the workshop; OBS, Observant (x3); “ConceptBoard” is an online platform which was used as an interactive discussion tool during these workshops.

Program part	Approx. Time	Content
Introduction	15	Welcome by MOD;
		Introduction of the workshop’s program and room for questions;
		Introduction and more information regarding the case “Genetic Engineering and the Rhizosphere” by MOD.
*Impact learning* Identifying Possible Issues	30	*In breakout sessions* in *ConceptBoard*:
		What issues do the participants foresee based on the case? Can be both positive (opportunities) and negative (possible risks). MOD and OBS1, OBS2 and OBS3 help structure the identified issues by grouping them.
*Break*		
*Normative Learning* Prioritizing Issues	30	*In breakout sessions* in *ConceptBoard*:
		MOD and OBS 2 help the participants to provide argumentation concerning the importance of the identified values based on relevant values;
		Results in a group of associated values;
		In four rounds, participants are asked to prioritize the earlier identified values in terms of importance. To do so, participants have to explain why they feel that a certain value is more important than another? Every round, each participant can move one value one level up, and one value one level down. This results in an illustration of how each value is prioritized (low importance—moderate importance—high importance)
		*Plenary* discussion is devoted to the outcomes of the breakout sessions.
*Institutional Learning*	30	A *plenary* discussion devoted to the following questions:
		*How to balance (uncertain) risks and (potential) benefits?*
		*How to establish norms for uncertain risks?*
		*Who should be responsible to ensure safety?*
		*To what extent is the current risk management system able to cope with the identified issues?*
Evaluation and Closure	15	MOD asks all participants what their take-home message is; Thank you to all participants and request for feedback; Closure of workshop.

Based on derived insights from the workshops conducted in March and June 2021, we developed the first set-up of the tool to enable an environment suitable for discussing and learning about uncertainties and uncertain risks. The workshops conducted in January and February 2022 were also dedicated to the validation of the tool, and therefore, these were slightly modified compared to the previous workshops. For instance, we decided to not use the interactive platform anymore as it turned out that participants were facing problems managing it in an online environment. Also, the workshops had more concrete steps which were: 1) identifying uncertainties and/or uncertain risks, 2) developing anticipatory strategies to lower or mitigate the earlier identified potential issues, and 3) determining what would be needed to implement the developed strategies in a researcher’s experimental set-up. Step 2—developing anticipatory strategies–adheres to the notion of Safe-by-Design (SbD), a promising iterative risk management approach to deal with potential risks of biotechnology applications by using materials and process conditions that are less hazardous (Bollinger et al., 1996; Khan and Amyotte, 2003; Robaey, 2018). This choice was based on providing the partaking stakeholders with more concrete guidelines for developing suitable strategies, which also came up during the evaluation of the first two workshops. Table 2 provides a short script of these workshops.

**TABLE 2:** Script for Workshops conducted in January and February 2022. MOD = Moderator of the workshop.

Program part	Approx. Time	Content
Introduction	30	Welcome by MOD;
		Introduction of the workshop’s program and room for questions;
		Introduction and more information regarding the case “Genetic Engineering and the Rhizosphere” by MOD.
Identification and Prioritization of Risks	20	*In breakout sessions*: Participants identify and discuss possible issues they foresee based on the case. In these groups, they try to come to a top-3, in which the possible issues are listed in order of importance (e.g., dependent on estimated severity or magnitude).
	15	*Plenary*: Each ‘break out group’ presents their top-3. Each group is invited to pose questions to the other.
*Break*		
Formulating Anticipatory Strategies	15	*In breakout sessions*: Participants discuss and develop SbD strategies that might lower or mitigate the earlier identified issues. MOD stresses that these measures do not all have to be technically oriented, but can also focus on e.g. procedural or organizational matters.
	15	*Plenary*: Each “breakout group” presents their strategies and explains how these would mitigate or lower the earlier identified issues. Each group is invited to pose questions to the other.
Identify Needs of a Researcher	20	This part revolves around the question: *What do researchers need to implement (the earlier identified) SbD strategies in their research?*
		Participants are given 5 min to put things in the chat. Subsequently, each participant is allowed to elaborate on the matters they’ve put in the chat.
		*Plenary* discussion about what of the listed matters are found most important—can we reach a consensus?
Evaluation and Closure	15	MOD asks all participants about their take-home message;
		Thank you to all participants and request feedback;
		Closure of workshop.

All conducted workshops provided insights into tensions and/or differing perspectives between stakeholder groups about the identification of uncertainties and uncertain risks, and what would be needed to anticipate or mitigate these. In response, themes were derived that helped clarify and structure these insights, of which a detailed overview is provided in Section 3. Section 4 elaborates on the utilization of the developed tool and to what extent this format can be used to initiate an active discussion between stakeholders about uncertainties and uncertain risks associated with emerging biotechnology applications.

### 2.2 Case: Genetic engineering in the rhizosphere

As already mentioned, in 2017, Hogervorst and colleagues pointed out several developments in the biotechnology field for which, at that moment, no adequate environmental risk assessment could be conducted. One of these developments is the genetic engineering of plants’ root exudates and their impact on the rhizosphere. The latter comprises the zone of soil around plants’ roots that is influenced by root activity and consists of micro-organisms that feed on sloughed-off plant cells, proteins and sugars released by the roots; the root exudates (Walker et al., 2003). By manipulating a plant’s root exudates, we can reduce our reliance on agrochemicals. Influencing the soil acidity in the plant root area can improve a plant’s productivity (Bais et al., 2006; Ryan et al., 2009). For example, in papaya and tobacco plants, researchers have overexpressed the enzyme citrate synthase which is responsible for the production of citric acid in the plant. This acid is excreted through the roots of the plant and causes an acidifying effect on the plant’s root zone. This effect can improve the availability of phosphate in the root zone, stimulating the plant’s growth. Also, it can cause partial alleviation of aluminum toxicity stress, a frequently occurring problem in soils that inhibits plant growth (De La Fuente et al., 1997).

The rhizosphere is a complex environment with plants, microbes, soil and climate conditions interacting. As many of these interactions are not yet well understood, performing an adequate risk assessment is impossible at the moment. Therefore, such genetic engineering approaches have never progressed beyond experiments demonstrating the proof of principle. However, recently, scientists noted that they believe CRISPR-Cas9-based genetic screening can help future studies of plant-microbiome interactions and discover novel genes for biotechnological applications (Barakate and Stephens, 2016). Also, others argue that new tools and resources can be applied to introduce complex heterologous pathways–that encompass both natural and biosynthetic routes–into plants. Such would allow for building synthetic genome clusters from microbiomes to enable stacking and shuffling of disease resistance and stress tolerance traits between crop plants (Shih et al., 2016).

At the start of each workshop, the case described above was introduced to all participants, which illustrated the dilemma of having insufficient knowledge about such a complex system while it is also a technique that has potentially great societal benefits such as improving the global food supply. This set the stage for the workshop and formed the starting point for an active discussion on how to manage associated uncertainties and uncertain risks safely and responsibly.

### 2.3 Participants

As genetic engineering in the rhizosphere is a case with high complexity, many interactions between variables and insufficient knowledge on many aspects, a broad variety of stakeholders were invited to take part in this workshop–see Table 3. The aim hereby was to retrieve a holistic approach to uncertainties associated with the case and to develop a range of anticipatory strategies to lower or mitigate these uncertainties while taking into account both impact, moral and institutional aspects of risk management.

**TABLE 3:** Participants’ Sectors and code.

Organization	
MOD	Moderator
OBS1	Observer
OBS2	Observer
OBS3	Observer/ Moderator
Workshop 16 March 2021	
RIT1	Research Institute—Technical Sciences
RIT2	Research Institute—Technical Sciences
RIT3	Research Institute—Technical Sciences
BSO1	Research Institute—Biosafety Officer
RIS1	Research Institute—Social Sciences
RO1	Regulatory Organization
RO2	Regulatory Organization
NG2	National Government
Workshop 3 June 2021	
RIT4	Research Institute—Technical Sciences
RIT5	Research Institute—Technical Sciences
RIS2	Research Institute—Social Sciences
RO3	Regulatory Organization
RO4	Regulatory Organization
Workshop 7 June 2021	
RIT6	Research Institute—Technical Sciences
RIT7	Research Institute—Technical Sciences
RIT8	Research Institute—Technical Sciences
RIS3	Research Institute—Social Sciences
RO5	Regulatory Organization
RO6	Regulatory Organization
Workshop 25 January 2022	
RIT9	Research Institute—Technical Sciences
RIT10	Research Institute—Technical Sciences
NG3	National Government
RO7	Regulatory Organization
BSO2	Research Institute—Biosafety Officer
RIS4	Research Institute—Social Sciences
Workshop 7 February 2022	
RIT11	Research Institute—Technical Sciences
RIT12	Research Institute—Technical Sciences
RIS5	Research Institute—Social Sciences
BSO3	Research Institute—Biosafety Officer
NG4	National Government
NG5	National Government
RO7	Regulatory Organization

A total of 32 stakeholders from a range of expertise participated in the workshops. Participants’ fields of expertise pertained to the technical sciences (i.e., microbiologists, biotechnologists, ecologists and Biosafety Officers), social sciences (i.e., (bio)ethicists, scholars working at the intersection of research and policy), regulatory organizations (i.e., risk assessors, policy officers) and the National Government (i.e., the Ministry responsible for national biotech regulation). We made sure that in every workshop a variety of stakeholders was partaking (see Table 3).

All participants were selected based on their knowledge of and/or experience with biotechnology applications and the regulation thereof. All hold senior positions in their designated professions, except for participant [RIT3] who was an MSc. Student Biotechnology and [RIT1] and [RIT6] were both PhD Candidates at the time of the workshop. Also, [RIS1] and [RIT12] are both professor emeritus. Lastly, MOD, OBS1, OBS2 and OBS3 were present in all workshops.

## 3 Results

All discussions in the workshops were transcribed, coded and analyzed in line with the three levels of “social learning” (see Section 2 Materials and Methods). These levels formed the three themes that need to be addressed to arrive at responsible learning about uncertainties. These themes are 1) Institutional learning entailing responsibilities, 2) Impact learning considering uncertainties and uncertain risks, and 3) Normative learning adhering to balancing uncertain risks with potential benefits. Furthermore, as part of the workshops was devoted to developing anticipatory measures, the notion of SbD was also discussed. However, as SbD is not considered the main focus of this paper, insights from these discussions are integrated into the other themes. Sections 3.1–3.3 elaborate on the tensions and differing perspectives between stakeholder groups in line with the identified themes. Section 3.4 provides an evaluation of the conducted workshops and to what extent these have led to social learning, and a summary of the “lessons learned.” These lessons functioned as input for the final design of the tool (i.e., the workshop script) which is elaborated in Section 4.

### 3.1 Institutional learning: Responsibility

The first identified theme revolves around responsibility concerning safety. With this, we refer to three matters; 1) researchers should apply a broad perspective on issues arising when developing a new technique or application thereof and taking anticipatory measures; 2) whether this should be done for both fundamental and applied research, and 3) unrealistic expectations concerning safety and the association with something being “natural” or not.

During the workshops, it became apparent that there is a consensus that researchers should make sure that their experiments are developed and conducted safely and responsibly. However, there were differences in how willing researchers would be to do so concerning possible long-term effects. On the one hand, participants [RIT9; RIS4; RO7] mentioned that researchers are probably not very willing to do so as they want to focus on answering the fundamental questions in research and generating new knowledge. In terms of long-term effects related to applications of their findings, stakeholders from other expertise might be better equipped to do so [RIT4].

“*The assumption here is somewhat that researchers want that too [talk and identify uncertainties], and I often find that very sobering when I speak to biotechnologists from [University], for example, who simply see that, that specific type of thinking is not their job at all. They mainly see the development of new knowledge as their task, and what risks there are is outsourced to, for example, [sub-department of University]. Or for a [regulatory organization] member.*” [RIS4]

“*I must also honestly say that I always try to keep myself a bit off from all the difficult follow-up things and think well, there are all [other] people who really like that and study bioethics, they can say useful things about it*” [RIT4]

Particularly in the light of the Asilomar Conference where researchers themselves took responsibility for ensuring the safe development of recombinant techniques (Berg et al., 1975; Berg and Singert, 1995; Abels, 2005), this was surprising. However, it was also argued that there certainly is a willingness amongst researchers, but tools need to be provided to do so [RIT10].

“*I do think that it is the researcher’s responsibility to think about this [emerging risks or other use than intended], not just the university’s. And I also think, on the one hand, there is some trust needed, that we [researchers] are certainly committed to... The whole purpose of the research we do is to make something better whether it's global health, the environment or whatever. So the benevolence is there. So, I need questions to be asked, for someone to point out a blind spot through a question, that makes me start to think about such. That’s what I need!*” [RIT10]

In terms of these tools, discussions in the workshops of January and February 2022 were devoted to SbD strategies mitigating or limiting identified risks. Researchers would probably bear the most responsibility to “do” SbD as they are working with emerging techniques, but that would require to know when this should be done [RIT11], and to what it specifically pertains [NG4]. Would that only be when an application is already foreseen, or also during fundamental stages of research [RO7]?

“*It is important, when should you do this? And certainly if you are an academic researcher you have a fundamental question. And should you immediately start applying SbD because an application may result from your research? And when should you build in those reflection moments? And how do you build it in?*” [RIT11]

“*I make sure that I work safely, so [I] protect myself as a researcher and then I’m working [in a] SbD [way]. But that’s not what we mean [with SbD]. But then you can say: when is it [SbD], and when is something not SbD? Does that mean you’re always improving [on safety]? Or will there come a point where you say: look, we’re here [it is safe enough]. Those are, I think, questions that are important for a researcher*” [NG4]

Some stakeholders pertaining to the social sciences domain argue that, from their perspective, researchers working on fundamental matters are not concerned with matters they consider outside their scope. For instance, [RIS4] argues that when researchers are working on a fundamental matter, this would be value-neutral from their perspective, and therefore there is no need yet to consider whether this would be a good or bad idea. Only in the next steps e.g., when there is an envisioned application “we will look at what harm can it do?” [RIS4]. However, this was nuanced by a participant from the technical sciences [RIT7] who argues that there are two stages “We try to understand the world and then we try to change the world, to make our lives better”. Thereby, [RIT7] acknowledges that applying insights one gained from *understanding* the world and trying to *modify* the world based on that knowledge are two different matters.

Also, there were discussions on what responsibility researchers have towards society in terms of communicating about risks and the meaning of safety–as biotechnology is still subject to public discourse. The discussion revealed two interpretations of safety, and how this is used and understood differently by different stakeholders. First of all, safety is often a technical matter in which a quantifiable chance of hazard (something that can cause harm) and how serious that harm could be is embedded–a definition that is frequently used by researchers from the technical sciences. However, the societal association with risks turns out to be ambiguous. Particularly in terms of risk communication, the societal interpretation of risk adheres more to the “absence of danger” [RO7; NG4]. In line with Beck et al. (1992), this association seems to be a response to society not being ‘in control’, but instead, organizations and governmental bodies responsible for the progress of biotechnological techniques and applications (Burgess et al., 2018). So, while technically safety refers to something having an acceptable risk involved, the societal interpretation is different.

“*Safety is also a concept defined by technicians, which is often where it comes from. And if we define safety as ‘the chance is so small that something will happen’ so we accept that, or we accept that because there is an advantage. But citizens understand safety as the absence of danger. So if you start talking about risks when you don’t even know if they are there - they are always there of course—But, then you already have a negative communication frame. And at the same time, you cannot guarantee safety*” [RO7]

“*So we as a government think that we cover everything with [acceptable] risks, but in principle, the citizen says ‘no, I want full protection’. Which of course is not realistic, you can never completely protect someone against something*” [NG4]

Also, there was some frustration detected in line with society’s stance on biotechnologies. Not necessarily due to safety concerns–whether that would be having an acceptable risk involved or by being “fully protected from danger”—but due to the association made with naturalness (de Graeff et al., 2022). And, in that respect, when something is “natural” that it would be safe(r). [RIT4] mentions that the distinction between what is natural and what is not has become a bit blurry. It is mentioned that putting a UV lamp on crops is still natural as it is just “…putting the sun on it a little harder” and people tend to think very quickly that “natural is safe”: “At least in the case when I talk to people about it, that’s the biggest difference. If it's natural, then you can sell it. If it's not natural, alarm bells will start ringing.” However, this association might be skewed as, given the recent pandemic, “corona is also natural and the vaccine we all receive is not natural” [RIT4].

### 3.2 Impact learning: Uncertainties and uncertain risks

Discussions also pertained to questions on appropriate strategies or measures to anticipate emerging risks, both short- and long-term. First of all, for short-term risks, strategies can be applied that limit possible risks. For example, one could ensure containment [RIT11; RIS5; RIT11; RIT1] or “that the plant is just one generation, or that you deprive the plant of the ability to reproduce” [NG4]. But for the long term, it might be a bit more difficult to understand issues arising and how to anticipate these properly. “So something is, typically in the lab, you will test something in the relatively short term, but we really rarely test for something in the long term. So there is lack of knowledge, usually for the long term effects” [RIT6]. On the other hand, participant [BSO3] mentions that taking heavy measures could be a strategy in itself to anticipate long-term risks. Lastly, [RIT11] questions how realistic this “testing for the long term” would be. In particular when a commercial party is involved: “How much time can you use to do this research? Especially if there is a commercial component to it. Ehm [sic], and that’s why I think that long-term effects are especially difficult to capture in research, so to speak. You will not have 50 years to study those effects!” [RIT11]

Also, there was discussion about anticipatory strategies mostly being risk avoidant. Although that would be a way to ensure safe research, it doesn’t solve the problem of learning about uncertain risks. Therefore, participants argued that there should be a distinction between strategies by which you aim to reduce uncertainties as much as possible, and strategies that make it possible to learn about the risks involved [RIS5]. However, some tension is expressed by stakeholders from the National government. On the one hand, although they prefer to choose the safest option from the start of a study, sometimes one does not know what the safest form is without researching it [NG4]. On the other hand, they [respective Ministry] are end-responsible for ensuring safety: “My role as a policy officer is to ensure that if something is genetically modified, it does not lead to a greater than a negligible risk to people, the environment and the living environment [sic]” [NG5] In that sense, learning about uncertain risks gives rise to a dilemma: ensuring safety and learning what the safest form is.

Following the discussion regarding strategies for avoiding risks and learning what uncertain risks entail, it was discussed whether uncertainties should always be regarded as uncertain risks, and when uncertainties can be deemed a risk. It was mentioned that there can be a knowledge gap in such instances which can create tension in risk management. For example, for one material we know that we lack very specific information concerning the long-term toxicity levels in humans. While for another material, we might not even know yet whether or not this would be toxic to humans in the short term. This higher degree of uncertainty illustrates that there are varying degrees of missing information regarding uncertainties. But, this does not mean that all uncertainties should already be considered an uncertain risk (van Asselt and Vos, 2006, van Asselt and Vos, 2008; Flage and Aven, 2015). This is also addressed by participant [RO7]: “Look, if we don’t know at all whether something has an effect, does it make sense to talk about risks? The fact that you say that there are risks, also means that you recognize that something is going on, and in this case, you don’t know that at all!” [RO7].

Furthermore, it is mentioned that with uncertain risks we tend to focus on “known unknowns.” However, given the vast pace of developments in the biotechnology field, it is expected that we will also have to deal with the “unknown unknowns” shortly–matters which we do not know yet. From a precautionary perspective, it would be justified to “keep our hand on the tap, and only open it when we know for sure what will come out!” [NG4]. Also, [RO2] mentions that as long you have insufficient data to obtain a proper view of the severity of risks, you should always assume the worst-case scenario. In other words: “if you don’t have all data to be sure that something does *not* happen, you should assume that this *will* happen so the risk assessment generates that you should be more careful with taking the next steps (i.e., from lab to environment)” [RO2]. However, it is also argued that the best way to deal with these upcoming uncertainties is to work together and organize the systems in such a way that we are equipped to deal with new uncertainties:

“*In other words, you should set up the systems in such a way that if those [new] uncertainties arise, that you all [technical scientists, social scientists, regulatory organization, national government] know and trust each other enough to find solutions together. And which one [new uncertainty] you will encounter is indeed unknown, but at least then you have the structure to do something with it*” [RIS4]

“*Yes, so gather more brainpower from different perspectives to get a clear picture of what those new risks [of the new uncertainties] are*” [RO7]

In terms of working together, participants discussed examples coming from other disciplines where organizations are collaborating to learn about uncertainties. For instance, participant [RIS4] referred to a study once conducted about antibiotic resistance that could be possibly passed on by micro-organisms, and [RO7] to the “safe-innovation approach” in the field of nanotechnology.

“*There was one study about antibiotic resistance that could possibly be passed on by micro-organisms. This actually showed that a researcher could not come up with the question that the employee [a risk assessor from a regulatory organization] asked him, [presumably] based on his [the researcher’s] own culture and knowledge and technological training. At the same time, that employee [from a regulatory organization] had no idea what was actually going on a fundamental, technical level of research. So in that [project’s] user committee, the two of them seemed to really hit it off and thought: “yes, you have [combined] knowledge, we can only answer this question [thoroughly] together!*” [RIS4]

“*Yes, I’m thinking now, that comes from the ‘nano-world’ That’s what they call *the safe-innovation approach*. I don’t know if it’s quite the same, but it is the commitment to … Let’s say, the innovator and the people who have risk knowledge, to bring them together faster, so that you can have that conversation [about uncertainties]. And then it's just a question of whether those two are good enough, or whether you should include even more perspectives? So that’s one of those thoughts that lives there and actually also in a protected environment, so to speak. So let’s say ‘Chatham House rules’ or something. That you can just talk openly without company secrets just being exposed on the table, so to speak.*”[RO7]

Lastly, participants mention that using nature as a threshold could help to indicate whether an uncertainty should be regarded as an uncertain risk. “To know whether something involves a risk, you should also try to compare it to already known, you know, similar cases. […] Also looking at what is already known about the type of changes that it might induce. And is that something that is already there in the environment?” [RO5]. However, discussions emerged about to what extent you could use nature as a reference, particularly if you are looking at a mechanism that is already present in nature, but that is also precisely the subject of intervention. “To what extent are they then [after intervention] comparable to mechanisms you find in natural systems?” [RIS5]. Also, how representative are tests performed under contained use? “For example, a soil in the greenhouse would already be tested there or say several soils: but how representative are they for the outside world, where it will eventually end up? It seems very complex to me to simulate a soil life and everything in the soil, so I think there is a modelling issue?” [NG3]. And, how desirable is it to mimic natural processes anyway? “Are natural processes always desirable and safe? So, is that always suitable to imitate? Nature has also developed enough dangerous situations and toxins, so what do we want to learn from nature and evolution?” [NG5].

### 3.3 Normative learning: Balancing risk and benefits

In all workshops, the potential benefits of developing technologies were mentioned and how these should be balanced with uncertain risks. In particular for emerging technologies where there is a (societal) benefit associated, emphasis is often placed on not being able to guarantee that something is safe [RO2]. Gene drive technology is discussed–a technological application with possible great societal benefits by for instance altering or eradicating disease-causing insects such as mosquitos. For such technologies, society seems to be reluctant to accept possible associated risks even though the benefit would be large [RO1]. For (bio)medical applications (red biotechnology), this balance seems different which can be mostly explained by to who the risks and benefits are attributed [RO2; RO7; NG3].

“*For health care, this balance would be different as the benefits and risks would all be for the same person*” [RO2]

“*And whose benefits are they?” [RO7] “Yes, whose benefits and whose risks?*” [NG3]

“*Of course, it's about whose benefits and whose risks it is, isn't it? So if the risks are for society, but the benefits are only for the [producer], then you have a different story than if it were equally divided. Then you have a different weighting framework*” [NG3]

So, there appears to be a difference in how society perceives the risks associated with red biotechnology, and therefore, there might be less societal scrutiny for this strand of biotechnology. From a regulatory perspective, this strand is also regulated differently (Bauer, 2002, Bauer, 2005; Abels, 2005) and benefits (e.g., a life-saving treatment) are included in the respective risk assessment. For white (industrial) and green (plant) biotechnology, benefits are not taken into account during the risk assessment [RO2]. However, according to [RIS1], there is always a risk-benefit analysis performed, albeit implicitly. “One continues with these [research/experimental] activities because there are benefits. So, in every risk assessment, benefits are underlying because why would we proceed with them if there weren’t any? So**,** implicitly there is always a risk/benefit weighing going on” [RIS1]. However, questions that emerged from this statement pertained to *who* makes, or should make, this (implicit) trade-off, and based on what information considering that the potential benefits are also uncertain. As justly mentioned by [NG3], “How should we account for these?” Following up on this remark, it was discussed that instead of trying to assign weight to the potential benefits and focusing on emerging risks, we could also look at what happens if we do nothing. For instance, related to the case, an expected benefit of engineering plants’ rhizosphere is contributing to improving the global food supply: “What happens when we don’t do it? Instead of well, just looking at what happens if we do it?”, and “Perhaps exactly by intervening we can maintain an existing ecosystem, while otherwise, we would lose it, for example. So not intervening with nature can also lead to a loss of biodiversity and so on” [RIT4]. However, other participants were sceptical of the ideas introduced by [RIT4]:

“*And there is also seldom talked about the uncertainties in advantages, it is always said: we can do this and it all yields this nicely. I’ve never heard of any uncertainty about the benefits*” [NG3]

“*No, the premise is usually it's going to save the world. As long as the risks are manageable, we will save the world!*” [RIS4]

Also, if we would include potential benefits in the risk-benefit balance, and therefore proceed with these technologies, we might eventually be able to improve the global food supply. But, this could give rise to new problems–perhaps no direct risks to one’s health, but more related to one’s livelihood and quality of life, i.e., economic and financial independence.

“*Suppose this becomes the staple crop in some country that normally doesn’t have such crops, say, will that displace other crops economically? Don’t know if you understand what I’m saying, I’ll give the example of Vanillin. You know, you can also do [produce] that with micro-organisms, but that means that in Madagascar suddenly less money is made with vanilla, and they suddenly have no income anymore. So that are other kinds of impact you could think about*” [RO7]

### 3.4 Evaluation and lessons learned

Based on the conducted workshops and the derived results presented in the previous sections, we first list the main findings and provide an evaluation of to what extent these workshops have contributed to social learning. Following upon, we formulate some “lessons learned” that formed the basis for the development of the final form of the tool to enable learning about uncertain risks which is presented in Section 4.

First of all, discussions associated with institutional learning entailed tensions about 3 matters: 1) responsibility allocation in the sense of researchers anticipating emerging risks, 2) whether these responsibilities should pertain to both short- and long-term risks and 3) apply to both fundamental and applied research. There was a consensus that ensuring safety is a responsibility that all associated stakeholders of an emerging technique or application should bear. In addition, researchers should be responsible to take anticipatory measures to lower or mitigate emerging risks, for instance through SbD. Based on this, some learning took place in the sense that participants are now aware of others’ stance and perception on allocating responsibilities. However, as no consensus was reached in terms of what responsibility should be assigned to which actor, we can conclude that the conducted workshops have led to limited learning in terms of institutional learning.

Secondly, impact learning has taken place in the sense that emerging uncertain risks and uncertainties associated with the case study were identified. For instance; “I think that for me the take home message is that, indeed, that SbD is looked at very differently.” And also: “When is something an uncertainty or a certain risk? And how should we as researchers deal with this?” [PIW], or: “So very often topics like this [the case study on engineering plants’ rhizosphere] don’t come up, but to participate in this discussion is certainly valuable. And if in the future, if these kinds of subjects become more topical for me, it will help me a lot” [RIT9]. However, participants were not able to come to a consensus in terms of the possible impacts or severity of these uncertainties. This could be due to the different fields of expertise of the partaking stakeholders, e.g. some having less technical insight into the possible effects of the identified uncertainties. Furthermore, participants agreed that researchers should be equipped with tools to be able to anticipate ‘new’ uncertainties. For instance, different stakeholders working together and reorganizing the internal system as the external system (i.e., GMO regulation with a strong emphasis on the PP) currently provides little room to conduct research with uncertainties or uncertain risks involved–which also reflects institutional learning.

Lastly, normative learning took place during the workshop as the participants gained insights into the dilemmas accompanying emerging technologies, and balancing their pros and cons. Particularly concerning the latter, the participants had to weigh the pros and cons associated with the case study to list what potential risks they considered the most severe or probable, and how to anticipate these. However, learning in terms of establishing new norms or reshaping the process of ERA did not take place. There were suggestions made and discussions devoted to these matters but without concrete results. While this could be partly explained by the partaking stakeholders having little influence on these matters (i.e., EU-level decisions), it can also be attributed to how the current regulatory system is operationalized, in particular in terms of the PP. Although this principle could stimulate learning (i.e., specifically setting up risk research as a precautionary measure) which is argued by the European Commission (Commission of the European Communities, 2000), it now provides a very normative approach to risks in the risk assessment system. This has resulted in a system that allows learning about known risks (albeit limited as there is already extensive knowledge of these risks) but only very limited learning what uncertain risks entail–depending on the extent of knowledge that is missing. Research involving uncertainties, thus having very little to no knowledge about the extent, is limited as it cannot be proven to be safe, i.e. having acceptable risks.

The main findings of the conducted workshops formed the basis for the development of a workshop format that enables a constructive discussion about emerging risks with a broad range of stakeholders. First of all, we should focus on researchers and provide them with tools to create a mutual learning environment to identify and anticipate emerging risks, and set up research devoted to learning what uncertain risks entail. An important condition for this, however, is that such discussions take place in an informal, non-institutional setting. This way, a truly free exchange of views and perspectives can take place without shared insights immediately having implications in terms of (societal) perception or in terms of (stricter or less strict) regulation. Fear of such consequences or implications can result in stakeholders keeping information or opinions to themselves. Such issues have already emerged in the (conventional) chemical industry, where there is little incentive for industry to share knowledge and data about possible adverse effects (Drohmann and Hernandez, 2020; Bouchaut et al., 2022).

Secondly, in the workshop format, SbD should not be specifically mentioned as this notion is understood differently by stakeholders. This was mentioned in the workshops and is also argued in literature (Bouchaut and Asveld, 2020; Kallergi and Asveld, 2021). We want researchers to have an open vision to develop anticipatory strategies to lower or mitigate identified risks. If we would mention SbD specifically, this could lead to a “tunnel vision” in which strategies would only pertain to e.g. technical measures. Also, it is important that stakeholders have a shared vocabulary, or that it is accommodated that stakeholders elaborate on what they specifically mean with certain jargon. During the workshops, there were sometimes misunderstandings between stakeholders when using e.g., technical terms or jargon from the policy or regulatory domain. Although such misunderstandings were addressed, and partaking stakeholders that needed some explanation did ask for this, it does illustrate that stakeholders must feel comfortable with each other. While this is a challenge, we expect this to become more feasible once discussions of these matters have become more common. Also, referring back to “new” uncertainties emerging in the (near) future, making such constructive discussions common practice will be good preparation to be able to deal with these accordingly.

## 4 Enabling stakeholder communication

Based on the lessons drawn from the conducted workshops (Section 3.4), we developed a final workshop format intending to enable a constructive discussion about emerging uncertain risks and to develop anticipatory strategies for ensuring safety. To do so, we chose the format of a protocol that facilitates researchers to organize a stakeholder workshop themselves. First, we envision the workshop to be organized by researchers who are working with (emerging) biotechnologies or biotechnological applications and invite researchers from other relevant areas of expertise such as ecology and toxicology, as well as stakeholders from the regulatory regime and other scientific disciplines such as (bio)ethics, social sciences and Biosafety officers. Thereby, it’s the intention that the organizing party composes a case of their own (as we have used genetic engineering of plants’ rhizosphere). For instance, the development of a new type of application or proceeding from a laboratory environment (contained) to a non- or semi-contained environment (e.g., field trials or clinical trials) where new uncertainties or uncertain risks can emerge. Secondly, by organizing this workshop, insights are gained into; 1) different estimates of uncertain risks, which risks are identified, on what basis, degree and nature of uncertainty, 2) defining anticipatory strategies to mitigate or lower the identified uncertain risks, and 3) determining what is needed to implement the defined strategy/strategies in their research practices.

Also, during the workshops and based on the evaluation with all partaking stakeholders, it turned out that there needs to be some incentive for researchers to place more emphasis on the identification and anticipation of risks (both short- and long term). Therefore, we would like to stress that this workshop brings value to researchers by not only ensuring safe and responsible research design but a greater emphasis on identifying and anticipating uncertain risks could also speed up research later in the process. For instance, when an experiment is initiated, additional information on possible risks may be required by an organization’s BioSafety Officer or a Member State’s respective GMO Office. Having already invested in a more extensive analysis of emerging risks, such processes might be accelerated or even prevented. However, it can also occur that a risk assessment (e.g., at the start of a new experiment) reveals that the experiment involves uncertain risks and that more data or research would be needed. This would also be a moment to initiate a workshop that would complete the risk assessment more thoroughly. Also, consultation with an organization’s BSO throughout the application process could create an incentive for organising this workshop. Therefore, we suggest researchers to organize this workshop when: researching emerging biotechnology applications; before composing or submitting a research proposal; when a risk assessment asks for extra information on emerging risks; and after consultation with an organization’s BSO.

**FIGURE 1:**
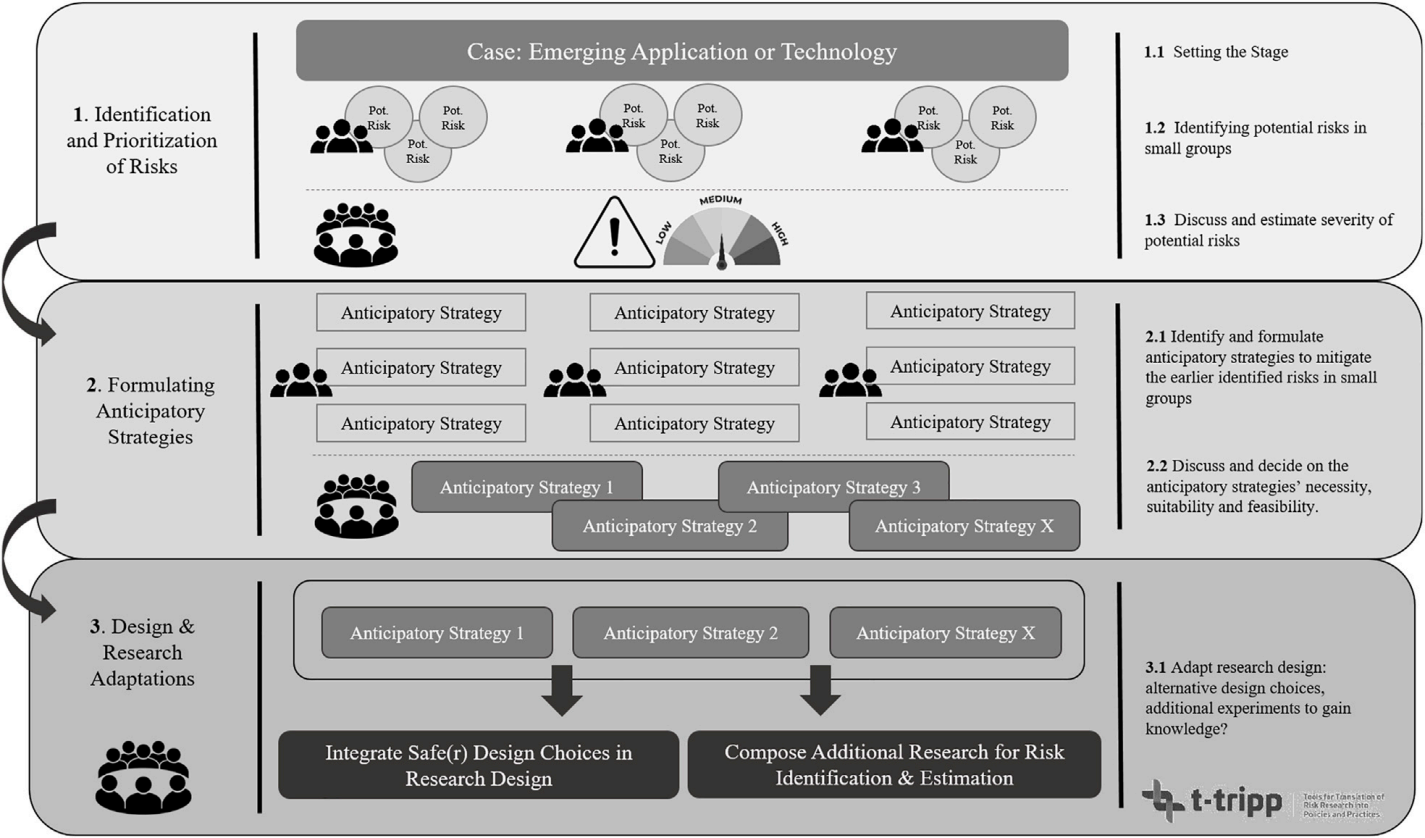
Flowchart for steps to follow during the social learning workshop.

A detailed script to organize this workshop is provided in Supplementary Appendix A, listing all preparatory measures for the workshop, organizational and practical matters e.g. hosting the workshop online or in a physical setting, and the elaborate steps that need to be taken for the execution of the workshop. For instance, one or multiple moderators need to be appointed as the workshop largely consists of discussions. In addition, we have created a flowchart (Figure 1) that schematically illustrates the protocol and briefly lays out the three main steps that need to be followed during the workshop. This flowchart can also be used by the organization to keep an overview during the workshop. The first step in Figure 1 entails the identification and prioritization of risks. Here, after the case is introduced at the beginning of the workshop, participants identify and discuss potential risks in small groups. Following upon, a plenary discussion is devoted to each group’s respective findings which are listed in terms of what potential risks are estimated the most important, which are considered less important, and why. In the second step, participants again discuss in small groups what anticipatory strategies could be applied to lower or circumvent the identified risks in step 1. The groups then return to a plenary setting in which participants decide on what strategies are considered the most effective, efficient, or suitable considering the research set-up. The final step is a plenary discussion devoted to discussing what would be needed to implement the earlier developed anticipatory strategies and whether these would lead to an acceptably safe research design. If not, the participants identify the knowledge gaps and how these could be filled in by setting up additional (risk) research.

## 5 Discussion

In this paper, we presented the development of a tool, i.e. a script for researchers, to organize a workshop to identify emerging risks and anticipatory strategies associated with emerging biotechnologies utilizing the notion of social learning and its three levels of learning about uncertainties (i.e., impact, normative and institutional). Also, integrating notions associated with the SbD-approach provides researchers insights into adaptations concerning their research design for increased safety and setting up additional risk research specifically for learning about “new” risks. The following aspects deserve attention as they have an influence on the execution and the outcomes of the workshop: 1) stakeholder representation, 2) free knowledge exchange and actors in bad faith, 3) expertise in moderating, observing and reporting, 4) the choice of the case, 5) the use of definitions and jargon, and 6) some limitations of our proposed method.

First of all, stakeholder representation is crucial for obtaining a holistic overview of any potential issues arising, and the extent to lower or mitigate these. For example, when specific techniques or applications with a geographically broad focus are discussed, the participants must have the experience and knowledge to discuss the case study in such a broad context. The organizers of the workshop must be aware of and should not underestimate the needed diversity of participants in order to arrive at a constructive, inclusive and broad discussion. As this workshop is tailored to biotechnology research and developments, it makes sense to especially invite stakeholders who are associated with the technical aspects related to this field. However, evaluations after our conducted workshops revealed that also the presence of social scientists and policymakers is crucial to arrive at safe biotechnology development beyond technical aspects and measures, and was even greatly appreciated by the partaking stakeholders from the technical sciences. Considering the set-up of our workshop, the organizers will be from the technical sciences, who might not have stakeholders from the regulatory or societal domain in their direct network. Therefore, identifying and inviting these stakeholders might take up some considerable time and calls for extra preparations, which must be taken into account by the organizing party.

Following upon, having an informal setting is needed to arrive at “free” knowledge exchange in which stakeholders from differing domains exchange their thoughts and experiences, and can pose critical questions. This is particularly relevant when working with a controversial technique or application. Therefore, inviting a wide range of stakeholders, including both proponents and opponents, is crucial to arrive at applications that will not be rejected by society (von Schomberg, 2013). However, knowledge exchange can also be exploited by actors who will attempt to block every process that does not fit the direction they desire. This places organizers in a difficult position. Whose input is considered valuable, and who to exclude from the discussion? As this allows for selectivity, it also gives rise to another misuse of the knowledge exchange processes, namely that researchers can choose to only invite stakeholders who fit exactly with their research aims.

As discussion is a key element of the workshops, the organizers must have considerable expertise in moderating, observing and reporting. Although we provide the methods to organize a workshop, the organizers are responsible for the execution and thus the outcomes. Therefore, we recommend having a moderator with a neutral stance on the discussed technology or application. While it can be advantageous that the moderator is affiliated with the same lab that is developing the discussed technique (i.e. having specific technical knowledge), we do not recommend this as this may result in bias. This also applies to the observer(s) and reporter(s).

Usually, a case will be highly specific to a certain technique–as was the case used in our workshops. While this brings focus to the discussion, one should be careful about subsequently generalizing the outcomes of the discussions. Also, due to the high specificity of the case, it may be difficult for some stakeholders to grasp the content as it’s not their field of expertise. On the other hand, the case being outside their “comfort zone” can also lead to obtaining new insights. Another issue concerns the timing of the introduction of the case to the participants. If already introduced before the workshop, the participants will be able to already think about the case and look up additional information. On the other hand, and also related to participants’ own field of expertise, they may decline the invitation as they feel that this would be beyond their expertise, thereby risking that valuable new insights will be missed.

Discussions in our workshops also revealed that there was some confusion in terms of used jargon and stakeholders’ definitions of e.g. uncertainty or risk, were not aligned. While having clear definitions is needed for effective communication, having differing definitions and interpretations can be used to shed light on stakeholders’ different perceptions of notions related to risks and uncertainties–which could also be valuable for the organizing party.

Finally, organizers should be aware that the method we present here also has limitations. First of all, the case used for the conducted workshops pertained to a highly complex environment. Although this contributed to making the dilemma clear of having insufficient knowledge, and continuing with promising developments, this may have caused some difficulties for participants to come up with concrete foreseen issues and anticipatory strategies. Secondly, in the case of the workshops we conducted, all stakeholders are associated with Dutch legislation. Although EU legislation is guiding, all EU Member States have their view on biotechnology and value different matters, and therefore, there might be a bias toward the Dutch perception. Thirdly, caution should be exercised when generalizing the outcomes of the workshop. Nevertheless, we believe that this tool is not only suitable to the field of emerging biotechnologies and can be used for other emerging fields such as nanotechnology or geo-engineering as well.

## Footnotes

1
https://doi.org/10.17026/dans-zta-6zz2
