# Supplementary material for: Uncertainties and uncertain risks of emerging biotechnology applications: A social learning workshop for stakeholder communication
Source: Front Bioeng Biotechnol. 2022 Sep 27;10:946526. doi: 10.3389/fbioe.2022.946526 (PMC9552295; doi:10.3389/fbioe.2022.946526)
Supplement: Supplementary file 1 [file DataSheet1.PDF]

Appendix A – Script for Workshop

As researchers find themselves at the cradle of emerging biotechnologies, it is important that safe and responsible development is ensured. By accommodating this workshop, researchers can see to it that this is done. This would be beneficial not only because safe and responsible development is highly endorsed by funding organisations, but also, in later experimental stages, it could prevent research to be delayed due to unforeseen issues arising, complicating matters such as conducting an adequate risk assessment and permit application. For instance, an issue could occur for which no policy is equipped yet. By identifying and anticipating these issues during early stages of experimental development, such delay or complication could be circumvented. Therefore, we provide a script that elaborates each step, the respective stages of thought to go through and the desired outcome or deliverable of each step. As mentioned, the ideal results of the workshop are a list of potential issues or uncertainties that need to be anticipated, suitable strategies to do so, and a list of design adaptations and/or requirements.

Eight notes of importance prior to the workshop:

1. First of all, when *inviting participants* it should be clear why they are invited for the workshop, how their specific contribution would be meaningful for the researchers and vice-versa (what's in it for them?). Thereby, the subject of the meeting and what will be discussed during the meeting should be clearly explained in the invitation.
2. As to avoid very general discussions about risks and uncertainties that lead to unclear recommendations, the subject of the meeting (i.e. the reason for organising this workshop) should be presented as a *specific case* that is in line with the (intended) research and forms the framework for the discussions. So, for instance, the development of a new type of application or proceeding from a laboratory environment (contained) to a non- or semi-contained environment (e.g. field trials) where new types of uncertainties can emerge.
3. The case should be sent to all participants prior to the workshop. Thereby, a balanced amount of information must be provided. It should be balanced in a way that experts can form a realistic idea of the different factors that may lead to new risks, and non-experts with less technical knowledge can place the 'case' in a broader picture.
4. Thirdly, it must be decided if an external *moderator* or *discussion leader* will be needed or whether one directly involved in the research project will act as discussion leader. We would recommend an external moderator due to the ability to act and summarize the discussions neutrally and who can create a positive and relaxed atmosphere. However, depending on the specific content the workshop will be focusing on, the moderator might require to have relevant (technical) knowledge considering the topic(s) for discussion. If this is not found to be possible, someone directly involved in the research project can act as discussion leader but should bear in mind to mostly focus on guiding, summarizing and reflecting on the discussion itself, instead of (perhaps subconsciously) letting one's opinion influence the discussions. Another option is to have a moderator that focuses on the discussions and have a 'second moderator' who focuses more on technical related matters. However, when choosing this option, the two moderators must make good arrangements of who answers/moderates what aspects.
5. Appoint a *rapporteur* with good reporting skills before the workshop for making a written report of the discussions.
6. Determine the *composition of break-out groups* (preferably balanced with different types of expertise in each group).

7. Make sure you are *well prepared* and have everything properly installed and tested: whiteboards, post-it notes, (felt-tip) pens, a camera to photograph results, tools for online support etc.
8. Lastly, if making recordings or if personal information will be used in the meeting's written report, ask participants for their permission using a *form of consent*[1]. This form of consent can be sent to the participants with the official invitation and should be handed in before the start of the workshop.

Script

In addition to the script, **Fig. 1** illustrates all steps to be followed and can be used as a supplementary tool during the workshop.

	Explanation	Outcome/ Deliverable
**Welcome**	The discussion leader welcomes all participants. Also, participants can be reminded of filling in the form of consent for making recordings during the workshop.	-
**Introductions**	All participants, including the discussion leader, shortly introduce themselves and indicate how they are involved with biotechnology and/or the relevant context under discussion.	-
**Step 1: Identification and Prioritization of Risks**		
**1.1 Introduction of aim/content workshop**	The discussion leader or researcher from the project introduces the program for the day and the aims of the workshop. Participants can ask questions regarding the aims, set-up or other details concerning the workshop.<br><br>Thereafter, the discussion leader or researcher from the project pitches the case on which the participants will focus during the workshop (point 2 above). Ideally, the case should be explained through several bullet points on a slide, thereby clearly stating the context (contained use or introduction to the environment, rationale of the research) and the central problem (complexities, uncertainties).	-

[1] For informed consent templates, see https://www.tudelft.nl/en/about-tu-delft/strategy/integrity-policy/human-research-ethics/informed-consent-templates-and-guide

	Participants can ask questions to clarify matters regarding the case.	
**1.2 Identifying potential risks**	Participants discuss in small groups (max 5 people in a physical setting and max 4 people when organised online) what possible risks are emerging according to their view or perspective. For this, participants are given 20 minutes to come to a consensus of 3 emerging issues, listed in order of importance.<br><br>Before the discussion starts, one of the participants should be appointed to make notes of the top-3 of potential issues. After the discussion in small groups, the lists will be discussed plenary.<br><br>For online settings, members of the research group can act as 'discussion leader' for the smaller groups to stimulate discussion and to provide more (technical) information when asked for by the participants.	A top-3 list (per group) of identified potential issues or uncertain risks
**1.3 Plenary discuss and estimate severity of potential risks**	Every group briefly presents their top-3 plenary. If groups weren't able to reach a consensus regarding a top-3, they should elaborate on the issues they ran into. Other participants can ask questions for clarification.<br><br>A plenary discussion (15 minutes) is devoted to the plausibility and severity of the identified issues, led by the moderator.	Overview of all listed potential issues.<br><br>Written report (by the rapporteur) with details concerning the estimated plausibility and severity of the identified potential risks, and an overview of issues that did not make the 'top-3' or was a lot of disagreement on.
**Step 2: Formulating Anticipatory Strategies**		
**2.1 Defining strategies**	Each small group (same composition as in step 1) discusses what anticipatory strategies they can think of for circumventing their top-3 of identified issues.<br><br>To stimulate or help discussion, the moderator can point out several technical strategies, e.g. kill-switches, auxotrophy, choice of an organism or implementing control mechanisms using, for instance,	List with anticipatory strategies for each respective group's top-3.

	light. In addition, other measures on (work)organisation can also be mentioned to spark discussion, e.g. proper lab training of staff. Again, one of the participants in each small group should be appointed to provide a summary in the plenary session that follows.<br><br>*Note*: one strategy can anticipate multiple possible issues. For more information and examples, see Robaey (2018).	
**2.2 Plenary discussion of anticipatory strategies**	Every group briefly presents their defined anticipatory strategies plenary. Other participants can ask questions for clarification.<br><br>A plenary discussion (15 minutes) is devoted to the effectiveness and feasible implementation of each strategy, and which would be the most effective to circumvent the earlier identified risks. Thereby, the defined anticipatory measures are also placed in the context of current regulation and legislation. Participants identify where there might be a lack of knowledge to adhere to the established norms to ensure safety.	Overview of all anticipatory strategies, and a list of which strategies are the most suitable.<br><br>Identification of knowledge gaps necessary to adhere to existing legislation and thereby ensuring safety.<br><br>Written report (by the rapporteur) with details concerning the estimated effectiveness and implementation of the defined strategies, and details concerning what strategy was deemed more suitable than another.
**Step 3: Design & Research Adaptations**		
**3.1 Formulating design adjustments**	First, participants are given 5 minutes to think of how the earlier identified strategies can be implemented in research. Or in other words, what would have to be adjusted in terms of the research design? For instance, there might be a need for more knowledge and/or additional risk research, more budget required for setting up the needed risk research, hiring extra staff, or more intense collaboration with the organization's BSO, etc.<br><br>Participants put their suggestions in the chat (online environment) or write them	Proposal for adjustments in the research design and complementary experiments specifically devoted to risk research.

	down for themselves (physical meeting). Following up, a plenary discussion is devoted to all suggestions made. The discussion leader addresses the participant's suggestions one-by-one, either from the chat or from what each participant has written down, and asks the participants to elaborate. Participants are encouraged to respond to each other's proposed adjustments.	
**Lessons learned and action points**	All participants share their thoughts about the workshop and its outcomes. Also, participants formulate a take-home message and suggestions for follow-up steps.<br><br>The rapporteur makes notes of all suggestions.	List with suggestions for follow-up steps and/or research.<br><br>Feedback from participants on the workshop and the outcomes.
**Summary workshop**	The discussion leader provides a recap of the workshop, and briefly summarizes the main outcomes of the meeting. Participants are allowed to respond and/or ask questions.	-
**Thank you & closure**	The discussion leader thanks the participants and concludes the workshop by summarizing how this workshop may contribute to adjusting the research (proposal).	-

Based on the outcomes of the workshop and the written report containing more detail concerning the discussions, the organizers of the workshop (i.e. the research consortium/PI/main applicants) should decide on what measures to take and implement them in their research design accordingly.
